# Non-Thermal Milk Decontamination by Ionic Modulation: A Deionization-Based Alternative to Pasteurization

**DOI:** 10.3390/foods15020387

**Published:** 2026-01-21

**Authors:** María T. Andrés, Jessica González-Seisdedos, Victoria Antuña, José F. Fierro

**Affiliations:** 1 Laboratory of Oral Microbiology (LMO), Clinical University of Odontology (CLUO), University of Oviedo, 33006 Oviedo, Asturias, Spain; andresmaria@uniovi.es (M.T.A.); jessica.gonzalez@madrid.org (J.G.-S.); uo282138@uniovi.es (V.A.); 2 Health Research Institute of the Principality of Asturias (ISPA), 33011 Oviedo, Asturias, Spain; 3 SamerLabs SL, Asturias Technology Park, 33428 Llanera, Asturias, Spain; 4 Department of Functional Biology (Microbiology), Faculty of Medicine, University of Oviedo, 33006 Oviedo, Asturias, Spain

**Keywords:** non-thermal milk processing, milk decontamination, ionic modulation, deionization, microbial safety, physicochemical quality

## Abstract

The dairy industry requires effective non-thermal processing strategies capable of ensuring microbial safety while preserving the nutritional and bioactive quality of milk. This study describes a novel milk decontamination approach based on selective ionic removal by dialysis, resulting in a controlled reduction in ionic strength. Milk deionization significantly reduced the microbial load in raw bovine milk to levels comparable to those achieved by conventional thermal pasteurization, while largely preserving its physicochemical composition. Ionic depletion enhanced the antimicrobial effectiveness of endogenous milk components; this effect was abolished when native salt concentrations were maintained, highlighting the key role of ionic modulation in microbial control. Major milk constituents, including proteins, fat, and solids-not-fat, were not substantially affected by deionization, whereas low-molecular-weight solutes such as lactose and urea were partially removed. Deionized milk also exhibited improved stability during refrigerated storage, as evidenced by delayed acidification compared with raw and pasteurized milk. Overall, these results demonstrate that milk deionization represents a feasible proof-of-concept non-thermal alternative to pasteurization based on ionic modulation, with potential applications in dairy processing and human milk preservation, where maintenance of bioactive components is particularly desirable.

## 1. Introduction

Milk is a nutritionally dense biological fluid containing proteins, lipids, lactose, and minerals, making it a fundamental component of the human diet worldwide [1,2]. These same attributes also make milk an excellent substrate for microbial growth, rendering it highly perishable if hygienic and storage conditions are inadequate. In raw milk, bacterial counts are generally low when milking is performed under aseptic conditions; however, contamination may occur rapidly through contact with animal surfaces (e.g., udders), human handling, or milking and storage equipment, thereby accelerating spoilage [3]. Refrigeration at 4–8 °C slows microbial proliferation, but microbial reduction remains essential to ensure milk stability and safety. Raw milk typically exhibits a pH of 6.6–6.8 and is commonly considered microbiologically unstable when values fall to ≤6.4 [4], a threshold associated with the onset of microbiological spoilage during refrigerated storage.

Human milk, considered the nutritional gold standard for infants and particularly important for preterm or low-birth-weight neonates, also requires strict microbiological safety to ensure pathogen-free administration [2]. Human milk may acquire exogenous microorganisms during collection, processing, or storage in milk banks, necessitating decontamination procedures that preserve nutritional and immunological quality [5]. Holder pasteurization (62.5 °C for 30 min), recommended by the World Health Organization and the European Milk Bank Association, remains the reference method despite its known limitations with respect to heat-sensitive bioactive components [6,7]. This need to balance microbiological safety with preservation of bioactivity has driven the search for alternative processing technologies [8,9].

Conventional thermal treatments—including low-temperature long-time (LTLT), Holder pasteurization, and high-temperature short-time (HTST) processing—are effective against vegetative pathogens but reduce the activity of heat-sensitive proteins such as immunoglobulins, lactoferrin, and lysozyme, as well as thermolabile vitamins [7,10,11]. Although sterilization extends storage stability, it markedly alters flavor and nutritional value [12]. In addition, heat-induced bacterial lysis has been reported to release endotoxins, which may pose potential risks, particularly for infants [13,14]. To overcome these limitations, several non-thermal preservation technologies have been investigated to ensure microbial safety while maintaining milk’s functional properties [15].

Physical non-thermal methods such as pulsed electric fields (PEF), ultraviolet (UV) irradiation, ultrasound, cold plasma, and high-pressure processing (HPP) have attracted increasing interest. Among these, HPP has been most widely implemented, as it effectively inactivates microorganisms while preserving thermolabile constituents [8,15]. Nevertheless, partial denaturation of lactoferrin and other immune components of milk under specific pressure–time conditions has been reported [16,17,18]. Synergistic approaches combining fermentation with emerging technologies (e.g., PEF, ultrasound, or cold plasma) have also shown potential to enhance microbial safety and remove undesirable compounds [19].

In contrast to direct microbial inactivation methods, membrane-based approaches achieve microbial control through selective separation. These include microfiltration, ultrafiltration, nanofiltration, and reverse osmosis, which enable protein fractionation, removal of microorganisms, and modulation of mineral content without the application of heat [8]. These technologies aim to extend shelf life and improve microbial quality while preserving bioactive components responsible for milk’s functional value [11,20].

Beyond physical processing, biopreservation strategies have exploited endogenous antimicrobial systems naturally present in milk, most notably the lactoperoxidase system (LPOS). This enzymatic system—comprising lactoperoxidase, thiocyanate, and hydrogen peroxide—generates reactive oxidizing species capable of inhibiting bacterial growth without heat treatment [21]. The documented effectiveness of LPOS under specific conditions highlights the potential of leveraging intrinsic milk components to enhance microbiological safety while preserving nutritional quality. This concept supports the rationale for exploring non-thermal approaches based on the functional activation of naturally occurring antimicrobial proteins, including lactoferrin.

Lactoferrin (Lf), a multifunctional transferrin-family glycoprotein, has been extensively characterized for its antimicrobial, antifungal, and antiviral properties [22,23]. However, its bactericidal activity in vitro is strongly inhibited by the presence of mono- and divalent cations [24,25]. These observations suggest that milk deionization may represent a strategy to activate the intrinsic bactericidal potential of lactoferrin, an approach that, to our knowledge, has not been systematically evaluated in milk systems. By lowering ionic strength and modifying the surrounding microenvironment, deionization may promote lactoferrin-mediated antimicrobial activity while largely preserving bioactive proteins and thermolabile components. This approach may be particularly relevant for human milk—which contains substantially higher levels of lactoferrin (~1–3 g/L) than bovine milk (~0.03–0.1 g/L)—provided it is appropriately validated for clinical use [26,27,28].

Previous studies have demonstrated that several endogenous antimicrobial components of milk, particularly lactoferrin, exhibit strong salt-dependent activity, which is markedly inhibited under physiological concentrations of mono- and divalent cations. This evidence suggests that modulation of ionic strength could represent an alternative route to microbial control. However, this concept has not been systematically evaluated in whole milk systems. Based on this rationale, we explored controlled ionic modulation via dialysis as a non-thermal milk decontamination strategy. The antimicrobial effects observed are discussed in the context of endogenous milk components whose activity is known to be influenced by ionic strength.

## 2. Materials and Methods

### 2.1. Sample Collection and Preparation

Raw bovine milk (*Bos taurus*) was obtained from clinically healthy cows approximately three years of age, with one or two calvings, from a family-owned dairy farm located in Illas (Taborneda, Spain). Milk was collected under hygienic milking conditions into sterile 50 mL polypropylene bottles and transported on ice to the laboratory for microbiological processing within 1 h of collection.

Each milk sample was divided into four equal aliquots and subjected to the following treatments: thermal pasteurization (low-temperature long-time, LTLT), static dialysis (deionization), dynamic continuous-flow dialysis (deionization), and untreated raw milk (control). All experiments were performed in triplicate for each condition.

Thermal pasteurization was performed by heating milk samples to 63 °C for 30 min in a thermostatically controlled water bath, as described in [29]. Temperature was continuously monitored to ensure stable conditions throughout the treatment. After pasteurization, samples were rapidly cooled to 4 °C and stored under refrigeration until further analysis.

### 2.2. Microbiological Analysis

Total aerobic bacterial load was quantified in raw, pasteurized, and deionized milk samples using the standard plate count method. Serial ten-fold dilutions were prepared in 10 mM Tris–HCl buffer (pH 7.4), and aliquots were plated on Milk Plate Count Agar (5 g/L casein enzymic hydrolysate, 2.5 g/L yeast extract, 1 g/L D-glucose, 1 g/L skim milk powder, and 15 g/L agar), following the Standard Methods for the Examination of Dairy Products [30]. Plates were incubated at 30 ± 1 °C for 48 h, after which colonies were enumerated and expressed as colony-forming units per milliliter (CFU/mL).

Bacterial viability in treated samples was compared with that in untreated controls to assess the effectiveness of the decontamination procedures. Species-level bacterial identification was performed using the automated VITEK^®^ 2 system (bioMérieux, Marcy-l’Étoile, France), which combines miniaturized biochemical tests with automated spectrophotometric detection.

### 2.3. Milk Deionization by Dialysis

Deionization was carried out using regenerated cellulose dialysis tubing (Spectra/Por^®^ 3, SpectrumLabs™, Los Angeles, CA, USA) with an internal diameter of 29 mm and a molecular weight cut-off (MWCO) of 3.5 kDa. Prior to use, dialysis membranes were conditioned according to the manufacturer’s instructions and rinsed thoroughly with ultrapure water (resistivity ~18 MΩ·cm; Milli-Q^®^ system, Millipore Corp., Bedford, MA, USA).

#### 2.3.1. Static Dialysis

Ten-milliliter milk samples were placed inside hydrated dialysis membranes and dialyzed at 4 °C. Distilled water was used as the dialysis medium to maximize ionic diffusion under controlled laboratory conditions, using a buffer-to-sample volume ratio of 200–500:1. Static dialysis was initially monitored for up to 12 h to determine the minimum time required to achieve ionic stabilization, as assessed by conductivity and ionic composition measurements. Dialysis was performed with ten water replacements at regular intervals. At the end of the process, dialyzed milk was collected aseptically and stored at 4 °C until further analysis.

#### 2.3.2. Dynamic Continuous-Flow Dialysis

Milk samples (10 mL) were placed inside dialysis membranes housed within an external impermeable chamber through which distilled water circulated continuously at a flow rate of 3 mL/min using a peristaltic pump (Masterflex^®^, Gelsenkirchen, Germany). Dynamic dialysis was initially monitored for up to 12 h to determine the kinetics of ionic removal. After treatment, dialyzed samples were collected aseptically and stored at 4 °C until use.

### 2.4. Determination of Deionization End Point

The time required to achieve effective deionization under each dialysis condition was determined using independent milk samples. Two analytical parameters were monitored:

(1) Electrical conductivity (µS/cm): measured using a calibrated conductivity meter (Crison^®^ Conductivity Meter Model 524, Hospitalet de Llobregat, Spain) with a 0.01 M KCl standard solution (1413 µS/cm) at 25 °C.

(2) Ionic composition (Na^+^, K^+^, Ca^2+^, Mg^2+^): quantified by inductively coupled plasma–mass spectrometry (ICP-MS) using an Agilent 7700X instrument (Agilent Technologies Inc., Santa Clara, CA, USA) equipped with an octopole collision/reaction cell and an Agilent I-AS autosampler.

Sample digestion and preparation were performed as previously described [31,32]. Deionization was considered complete when two consecutive measurements of conductivity or ionic concentration showed no further decrease.

### 2.5. Physicochemical Analysis

Aliquots collected before and after dialysis were analyzed to assess potential changes in milk composition. Physicochemical parameters, including fat, total protein, lactose, urea, total solids, and somatic cell count, were determined following standardized ISO procedures. All experiments were performed in triplicate for each condition.

Milk composition was analyzed by mid-infrared (MIR) spectroscopy using a MilkoScan™ FT/IC instrument (Foss Electric A/S, Hillerød, Denmark), calibrated according to the following reference methods: protein (ISO 8968-2/IDF 20-2), fat (ISO 1211/IDF), lactose (ISO 26462/IDF 214), and total solids (ISO 6731/IDF 21). Prior to MIR analysis, samples were homogenized and heated to 40 °C for 20 min.

Somatic cell counts (SCC) were mined by fluorescence-based flow cytometry using a Fossomatic™ analyzer (Foss Electric, Hillerød, Denmark), following ISO 13366-2/IDF 148-2:2006, which quantifies stained DNA signals emitted as individual cells pass through a laser beam.

### 2.6. Shelf-Life Estimation

The stability of deionized milk during refrigerated storage was evaluated by monitoring pH changes at 4 °C over a 10-day period, in parallel with raw and pasteurized milk samples. Daily aliquots were withdrawn under aseptic conditions, and pH was measured using a calibrated pH meter (Crison^®^ micro-pH 2000, Hospitalet de Llobregat, Spain) equipped with a combined pH 5233 electrode. pH values ≤ 6.4 were considered indicative of microbiological spoilage.

### 2.7. Statistical Analysis

Results are expressed as mean ± standard deviation (SD) from at least three independent experiments. Statistical analyses were performed by one-way ANOVA followed by Tukey’s post hoc test for multiple comparisons using GraphPad Prism version 10.2.2 (GraphPad Software Inc., San Diego, CA, USA). Differences were considered statistically significant at *p* < 0.05.

## 3. Results

### 3.1. Determination of Deionization Time

Because the minimum time required for effective milk deionization was not known a priori, conductivity and ionic composition were monitored over an extended period (up to 12 h) for both static and dynamic dialysis systems.

**Figure 1:**
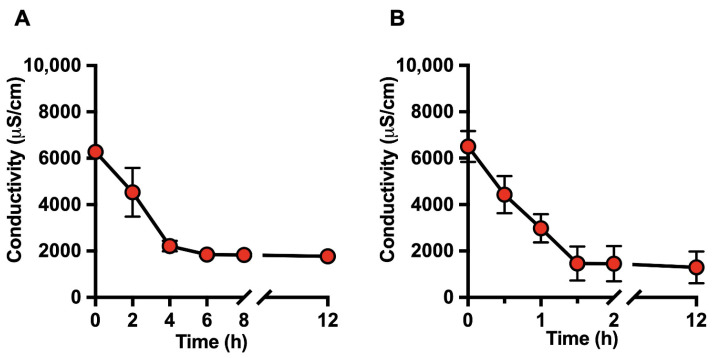
Time-dependent evolution of milk electrical conductivity during deionization by dialysis. Electrical conductivity (µS/cm) of raw bovine milk was monitored over a 12 h period during (**A**) static dialysis and (**B**) dynamic continuous-flow dialysis as an indicator of ionic removal. Data are expressed as mean ± SD of at least three independent experiments.

Figure 1A,B show the time-dependent decrease in milk electrical conductivity over a 12 h dialysis period using static and dynamic continuous-flow systems, respectively. Under static dialysis conditions, milk conductivity progressively decreased, reaching an approximate reduction of 70% after 6 h (Figure 1A). In contrast, a comparable reduction was detected much earlier in the dynamic continuous-flow dialysis system, with conductivity values decreasing by approximately 70% within 1.5 h (Figure 1B). In both systems, conductivity values remained stable thereafter, indicating that ionic equilibrium had been reached.

To further characterize the deionization process, the concentrations of major milk cations (Na^+^, K^+^, Ca^2+^, and Mg^2+^) were monitored and expressed relative to their initial levels in raw milk, as detailed in Table 1, which were consistent with previously reported literature values [4,33]. These ions were selected because of their documented ability to interfere with the antimicrobial activity of lactoferrin. Achieving a marked reduction in these cationic species was therefore required to establish a low-ionic-strength environment.

**Figure 2:**
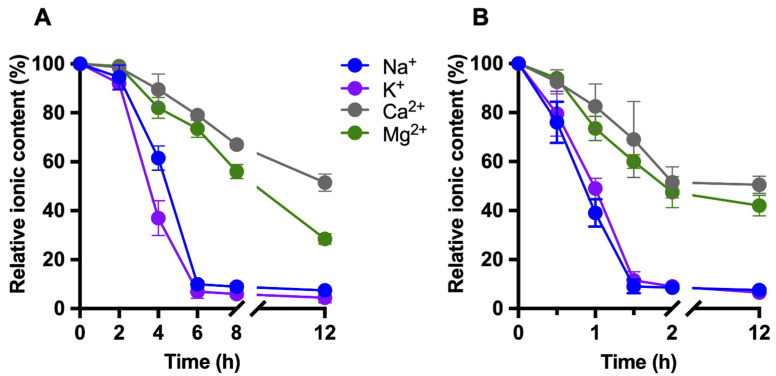
Time-dependent relative changes in milk ionic composition during deionization by dialysis. Concentrations of Na^+^, K^+^, Ca^2+^, and Mg^2+^ were determined by ICP-MS at different time points during (**A**) static dialysis and (**B**) dynamic continuous-flow dialysis and expressed as percentages relative to their initial values (t = 0 h, 100%). The x-axis includes a break to emphasize early dialysis kinetics. Data are shown as mean ± SD (*n* ≥ 3).

The time-dependent evolution of relative ionic concentrations during static dialysis is illustrated in Figure 2A. A progressive, ion-dependent decrease was detected, with the most pronounced reductions occurring during the first 6 h of treatment. Monovalent cations (Na^+^ and K^+^) exhibited faster removal kinetics than divalent cations (Ca^2+^ and Mg^2+^). In the dynamic continuous-flow dialysis system, minimum ionic concentrations were reached considerably earlier. As shown in Figure 2B, two consecutive samples collected at 1.5 and 2 h displayed comparable minimal ion levels, indicating completion of the deionization process under these conditions.

Overall, the decrease in milk electrical conductivity closely correlated with the time-dependent removal of mono- and divalent ions, particularly Na^+^ and K^+^, as determined by ICP-MS analysis (Figure 1 and Figure 2). The stabilization of both conductivity and ionic profiles was considered indicative of effective deionization. Based on these results, dialysis times of 8 h for static dialysis and 2 h for dynamic dialysis were selected as optimal end points for subsequent analyses.

### 3.2. Reduction in Microbial Load in Deionized Milk

Microbial load was assessed once stable deionization conditions had been reached, corresponding to 8 h for static dialysis and 2 h for dynamic dialysis. Total aerobic bacterial counts were determined using the standard plate count method.

**Figure 3:**
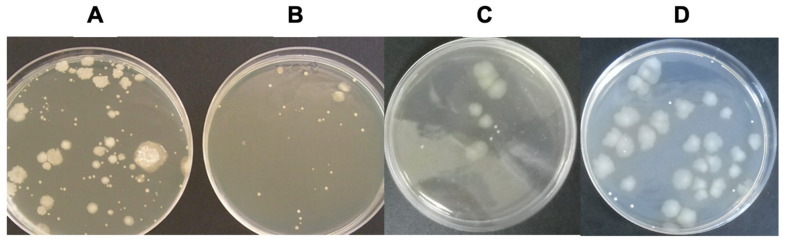
Representative microbial growth patterns in raw, pasteurized, and deionized milk samples. Bacterial growth obtained from serial dilutions of milk samples collected (**A**) before treatment (raw milk), (**B**) after thermal pasteurization, (**C**) after static deionization, and (**D**) after dynamic deionization. Total aerobic bacterial counts were determined using the standard plate count method.

As shown in Figure 3, a marked reduction in bacterial growth was detected in milk samples subjected to both deionization procedures compared with untreated raw milk. Plate counts revealed a substantial decrease in the number of viable microorganisms following both static and dynamic dialysis.

Quantitative analysis confirmed a significant reduction in total aerobic bacterial counts in deionized milk samples obtained by both dialysis approaches, with decreases of approximately 70% relative to untreated raw milk (Table 2). Although pasteurized milk showed slightly lower mean aerobic counts, no statistically significant differences were observed between pasteurized and deionized milk samples (*p* > 0.05), as confirmed by one-way ANOVA followed by Tukey’s post hoc test.

### 3.3. Microbial Profile of Deionized Milk Samples

Bacterial identification performed using the automated VITEK^®^ 2 system revealed the presence of microorganisms commonly associated with milk handling and environmental contamination in raw milk samples, including *Escherichia coli*, *Kocuria kristinae*, *Kocuria varians*, *Sphingomonas paucimobilis*, *Staphylococcus hominis*, *Staphylococcus warneri*, and *Yersinia enterocolitica*. Spoilage-associated *Pseudomonas* spp. were also detected.

Following deionization, the residual microbial population consisted mainly of environmental and spoilage-associated Gram-positive species. Gram-negative bacteria such as *Pseudomonas* spp., detected in raw milk, were not recovered after either static or dynamic dialysis treatments. Several Gram-positive genera, including *Bacillus* spp. and *Kocuria* spp., were identified in deionized samples.

### 3.4. Influence of Ionic Strength on the Antibacterial Activity of Deionized Milk

The natural concentration of lactoferrin in bovine milk is sufficient to exert antimicrobial activity under low-ionic-strength conditions; however, this activity is known to be inhibited by sodium and other cations. To evaluate the influence of ionic strength on the antibacterial effect observed in deionized milk, dialysis experiments were conducted under conditions in which ionic strength was maintained.

**Figure 4:**
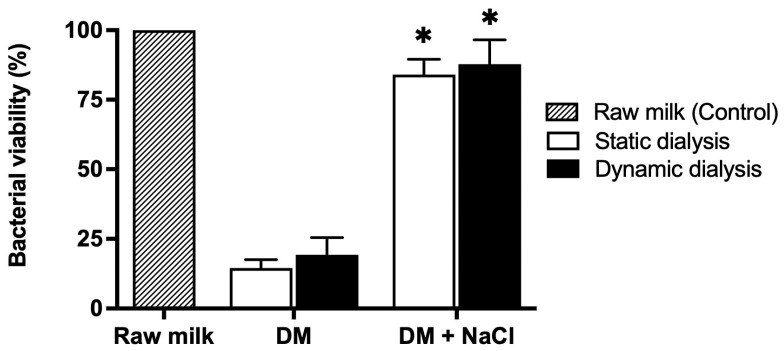
Effect of NaCl on the antimicrobial activity of deionized milk. Percentage of viable bacteria determined by the plate count method in raw milk (hatched bar, control), deionized milk (DM) obtained by dialysis in the absence of salt, and milk dialyzed against a NaCl-containing solution (DM + NaCl). Bacterial viability is expressed relative to raw milk (set to 100%). Data are presented as mean ± SD (*n* ≥ 3). * *p* < 0.05 compared with deionized milk (DM) in the absence of salt.

Milk samples were dialyzed for 8 h against a dialysis solution containing NaCl at a concentration of 25.5 mM, corresponding to the sodium levels measured in raw milk. Under these conditions, no significant reduction in total aerobic bacterial counts was detected. As shown in Figure 4, microbial loads in milk samples dialyzed in the presence of salt were comparable to those detected in untreated raw milk and markedly higher than those measured in milk dialyzed in the absence of salt.

These results indicate that maintenance of ionic strength counteracted the antibacterial effect associated with milk deionization.

### 3.5. Comparative Effects of Decontamination Processes on Milk Composition

Analysis of milk composition showed that deionized milk largely retained a physicochemical profile comparable to that of raw milk, with only moderate variations observed for specific parameters. Data in Table 3 are presented as mean ± standard deviation, and statistical comparisons were performed using raw milk as the control condition.

The slightly higher somatic cell count observed in pasteurized milk is consistent with previous reports and is attributed to heat-induced alterations affecting fluorescence-based cell counting rather than a true biological increase in cell number [34]. Minor variations in fat content are also likely due to heat-induced changes in fat globule structure and analytical variability [4].

Protein and fat contents in deionized milk remained within the ranges observed for raw milk, showing no substantial alterations as a result of the deionization process. Similarly, solids-not-fat (SNF) values in deionized milk were comparable to those of raw and pasteurized milk. In contrast, lactose concentration exhibited a noticeable decrease in deionized milk compared with both raw and pasteurized samples; urea showed a similar decrease following deionization, consistent with the removal of low-molecular-weight, dialyzable components.

Somatic cell counts in deionized milk were similar to those detected in raw milk, indicating that the deionization process did not adversely affect this parameter. A slightly higher somatic cell count was detected in pasteurized milk (Table 3).

### 3.6. Monitoring of Microbial Spoilage During Refrigerated Storage

The stability of deionized milk during refrigerated storage was assessed by monitoring pH changes over a 10-day period at 4 °C, in parallel with raw and pasteurized milk. The initial pH of all samples was approximately 6.7.

Raw milk exhibited a progressive decrease in pH, reaching values ≤ 6.4 by day 4 of storage. Pasteurized milk showed a slower acidification profile and maintained pH values ≥ 6.4 until day 5. In contrast, milk deionized by static dialysis maintained pH values ≥ 6.4 until day 7.

These results indicate that deionized milk displayed delayed acidification compared with raw and pasteurized milk under the conditions tested.

## 4. Discussion

The dairy industry faces the ongoing challenge of ensuring microbiological safety while preserving the nutritional and functional quality of milk. Conventional thermal treatments, including pasteurization and ultra-high-temperature processing, are effective in reducing microbial load but are also associated with degradation of heat-sensitive bioactive components, particularly proteins with immunological and antimicrobial functions [35,36]. Lactoferrin is a notable example, as its denaturation temperature overlaps with the temperature range used during thermal decontamination, resulting in a substantial loss of biological activity [37].

In the present study, a non-thermal, proof-of-concept strategy based on controlled ionic modulation was applied to reduce the microbial load of milk through deionization by dialysis. This approach achieved microbial reductions comparable to those obtained by conventional thermal pasteurization, while largely preserving the physicochemical characteristics of raw milk. These findings support the feasibility of ionic modulation as a potential alternative means of microbial control in dairy systems.

Previous studies have demonstrated that lactoferrin exerts direct antimicrobial activity in vitro through inhibition of the plasma membrane H^+^-ATPase, leading to cytoplasmic acidification that overwhelms cellular homeostatic mechanisms and ultimately results in cell death [23,38,39]. However, this activity is strongly inhibited by the presence of mono- and divalent cations [24,39,40]. Mono- and divalent cations are known to stabilize bacterial membranes and reduce electrostatic interactions between lactoferrin and the microbial surface. Hypothetically, ionic depletion lowers membrane stabilization and facilitates lactoferrin binding, resulting in disruption of ionic homeostasis and loss of cellular viability [23,24]. Taken together, these mechanisms provide a coherent biological basis for the enhanced antimicrobial activity observed under low-ionic-strength conditions in whole milk.

Bovine and human milk naturally contain sodium and potassium concentrations that are sufficient to markedly reduce this activity [33]. The objective of milk deionization in this study was therefore to reduce ionic concentrations to levels comparable to those used in vitro, in order to assess whether ionic depletion in a complex biological fluid could activate the salt-sensitive antimicrobial action of lactoferrin. During dialysis, cation removal was not uniform, reflecting the distinct distribution of ions between the soluble and colloidal phases of milk. Monovalent cations such as Na^+^ and K^+^ were almost completely removed, consistent with their presence as freely diffusible salts, whereas Ca^2+^ and Mg^2+^ exhibited a more limited decrease due to their partial association with casein micelles and milk proteins [4,41].

Consistent with this ionic depletion, deionized milk exhibited a marked reduction in cultivable microbial load, likely reflecting enhanced lactoferrin-mediated antimicrobial activity favoured by low-ionic-strength conditions. Notably, several Gram-negative bacteria detected in raw milk, including *Escherichia coli*, *Pseudomonas* spp., and *Yersinia enterocolitica*, were no longer detectable following deionization. These observations are consistent with the known susceptibility of Gram-negative bacteria to lactoferrin-mediated antimicrobial activity under low-ionic-strength conditions. In contrast, some Gram-positive species persisted after treatment, suggesting differential sensitivity to ionic depletion and lactoferrin activity, as previously reported for other antimicrobial peptides. This study focused on total aerobic counts as a primary indicator of microbial load. From a regulatory perspective, the microbial reductions observed in this study should be interpreted as indicative of pasteurization-equivalent performance at the level of total aerobic counts, but not as formal validation according to dairy safety regulations [42]. Formal regulatory equivalence was beyond the scope of this proof-of-concept study and requires pathogen-specific and indicator-based testing (e.g., coliforms), which will be essential in future studies designed for industrial and clinical validation.

Further evidence for the role of ionic strength in the observed antimicrobial effect was obtained by maintaining sodium concentrations during dialysis. When milk was dialyzed against a NaCl-containing solution at concentrations similar to those found in raw milk, no significant reduction in microbial load was detected. This finding indicates that preservation of ionic strength effectively counteracted the antibacterial effect associated with deionization and supports the involvement of salt-sensitive endogenous antimicrobial mechanisms. Although lactoferrin is the most likely contributor, the involvement of additional antimicrobial components and/or conditions cannot be entirely excluded.

Beyond microbial safety, milk deionization largely preserved the physicochemical composition of milk. Major constituents such as protein, fat, and solids-not-fat remained within the ranges observed for raw milk, indicating that the dialysis process did not adversely affect these components. Similar preservation of macronutrient composition has been reported for other non-thermal technologies, such as high-pressure processing [43,44]. In contrast, the apparent variations observed in pasteurized milk for fat content and somatic cell count are likely attributable to analytical artifacts induced by heat treatment rather than true compositional changes [4,34].

A pronounced reduction in urea content was evident in deionized milk. From a dairy processing perspective, this reduction may be advantageous, as urea has no direct nutritional value and can negatively affect processing performance and product quality [45,46]. As expected for a membrane-based process, deionization also resulted in partial removal of low-molecular-weight solutes, including lactose. Similar effects have been reported for microfiltration and ultrafiltration processes [8,47] and highlight the need for controlled post-processing restoration to maintain optimal nutritional and sensory properties. At the same time, membrane-based processes may offer the additional advantage of removing undesirable low-molecular-weight contaminants, such as antibiotics, pharmaceutical residues, mycotoxins, and hormones. Indeed, membrane filtration has been shown to eliminate commonly used veterinary antibiotics (Mw ≤ 1 kDa) and steroid hormones (Mw 270–400 Da), well below the dialysis membrane cut-off [48,49,50].

In addition to reducing microbial load, deionization delayed acidification during refrigerated storage, indicating improved microbial stability compared with both raw and pasteurized milk. This extension of shelf life is consistent with the observed reduction in viable microorganisms and further supports the potential of ionic modulation as a preservation strategy.

To date, very few studies have specifically examined milk deionization as a strategy for microbial control [51]. Most available literature on mineral reduction in dairy systems focuses on demineralization of whey or milk-derived streams for technological or nutritional purposes rather than microbial safety. Consequently, direct comparisons with previous deionization-based milk decontamination studies are limited, and the present work should be regarded as a proof-of-concept within this emerging research area.

Previous studies have shown that non-thermal technologies, including high-pressure processing and membrane filtration, can extend the refrigerated shelf life of milk while preserving heat-sensitive bioactive components. The delayed acidification observed in deionized milk in this study is consistent with these reports and reflects reduced microbial activity during storage. However, unlike conventional membrane processes, deionization does not aim to physically retain or exclude microorganisms but instead modifies the ionic environment to suppress microbial growth.

Together, these findings position milk deionization as a complementary non-thermal preservation strategy that differs mechanistically from previously reported approaches and may offer particular advantages where preservation of bioactive milk components is desirable.

Membrane-based processes such as microfiltration and ultrafiltration are already employed in the dairy industry but are often limited by membrane fouling and high operational costs. In contrast, the deionization approach described here relies on activation of intrinsic antimicrobial mechanisms rather than direct microbial removal or inactivation. This characteristic positions milk deionization within the broader framework of biopreservation and sustainable food processing. Importantly, the water consumption associated with laboratory-scale dialysis is not directly extrapolable to industrial implementation. Industrial deionization technologies rely on continuous-flow systems, electrodialysis, and recirculation strategies that drastically reduce water demand. Therefore, sustainability considerations should be evaluated in the context of energy efficiency and preservation of bioactive components rather than absolute water usage at laboratory scale.

Taken together, these results indicate that milk deionization represents a promising non-thermal alternative to conventional pasteurization, capable of improving microbial safety while largely preserving the physicochemical and functional properties of milk. Although controlled restoration of small solutes is required following dialysis, ionic modulation offers a low-energy strategy with potential applications in dairy processing and human milk preservation, where maintenance of bioactive components is particularly desirable.

## 5. Conclusions

This study demonstrates that milk deionization based on controlled ionic modulation achieved reductions in microbial load comparable to thermal treatment, while largely preserving the physicochemical properties of milk and enhancing stability during refrigerated storage.

These findings identify ionic strength as a key processing parameter influencing the antimicrobial effectiveness of endogenous antimicrobial systems in milk. Selective reduction in ionic concentrations through deionization appears to promote salt-sensitive antimicrobial mechanisms without the application of heat, supporting its potential as a mild preservation strategy. Future work should focus on process intensification, sensory acceptability, regulatory validation, and integration of ionic modulation into existing dairy processing workflows.

## Figures and Tables

**Table 1:** Major cation concentrations in raw bovine milk prior to deionization. Baseline concentrations of Na^+^, K^+^, Ca^2+^, and Mg^2+^ in raw cow’s milk determined by ICP-MS. Values are expressed as concentration ranges in mg·kg^−1^ and mmol·kg^−1^.

Cation	Concentration	
	mg·kg^−1^	mmol·kg^−1^
Na^+^	494–597	21.5–26.0
K^+^	1239–1528	31.7–39.1
Ca^2+^	1092–1223	27.2–30.5
Mg^2+^	103–138	4.2–5.7

**Table 2:** Aerobic bacterial counts in raw, pasteurized, and deionized milk samples. Total aerobic microbial load was quantified by the standard plate count method and expressed as colony-forming units per milliliter (×10^3^ CFU/mL). Values represent mean ± SD of at least three independent experiments. Different superscript letters indicate statistically significant differences (*p* < 0.05).

Decontamination Procedure	Microorganisms (×10^3^ CFU/mL ± SD)
Untreated (raw milk)	3.9 ± 0.2 ^a^
Pasteurization	1.8 ± 0.4 ^b^
Static dialysis	1.3 ± 0.1 ^b^
Dynamic dialysis	1.5 ± 0.3 ^b^

**Table 3:** Physicochemical composition of raw, pasteurized, and deionized milk samples. Milk components were determined by mid-infrared (MIR) spectroscopy using a MilkoScan™ FT/IC analyzer. Values are expressed as mean ± standard deviation. Different superscript letters within the same row indicate statistically significant differences (*p* < 0.05). % m/m, mass percentage; SNF, solids-not-fat; SCC, somatic cell count.

Milk Component	Raw Milk	Pasteurized Milk	Deionized Milk
Protein (% m/m)	3.83 ± 0.08 ^a^	3.44 ± 0.19 ^b^	3.60 ± 0.12 ^ab^
Fat (% m/m)	3.45 ± 0.25 ^a^	3.70 ± 0.20 ^a^	3.30 ± 0.20 ^a^
SNF (% m/m)	9.43 ± 0.24 ^a^	8.75 ± 0.18 ^b^	8.97 ± 0.13 ^b^
Lactose (% m/m)	4.83 ± 0.06 ^a^	4.74 ± 0.04 ^a^	3.68 ± 0.03 ^b^
Urea (mg/kg)	337 ± 15 ^a^	285 ± 3 ^b^	44 ± 2 ^c^
SCC (×10^3^ cells/mL)	158 ± 2.5 ^a^	166 ± 1.7 ^b^	147 ± 1.3 ^a^

## Data Availability

All relevant data are within the manuscript and are available upon request from the corresponding author.

## Abbreviations

CFUs	Colony-forming units
HPP	High-pressure processing
HTST	High-temperature short-time
ICP-MS	Inductively coupled plasma–mass spectrometry
Lf	Lactoferrin
LPOS	Lactoperoxidase system
LTLT	Low-temperature long-time
MIR	Mid-infrared
MWCO	Molecular weight cut-off
PEFs	Pulsed electric fields
SCC	Somatic cell count
SD	Standard deviation
SNF	Solids-not-fat
UV	Ultraviolet
